# Prolonged repolarization in the early phase of ischemia is associated with ventricular fibrillation development in a porcine model

**DOI:** 10.3389/fphys.2023.1035032

**Published:** 2023-01-23

**Authors:** Olesya G. Bernikova, Alena S. Tsvetkova, Mikhail A. Gonotkov, Alexey O. Ovechkin, Marina M. Demidova, Jan E. Azarov, Pyotr G. Platonov

**Affiliations:** ^1^ Department of Cardiac Physiology, Institute of Physiology, Komi Science Center, Ural Branch, Russian Academy of Sciences, Syktyvkar, Russia; ^2^ Department of Mathematical Physiology, Institute of Immunology and Physiology, Ural Branch, Russian Academy of Sciences, Ekaterinburg, Russia; ^3^ Institute of Medicine, Pitirim Sorokin Syktyvkar State University, Syktyvkar, Russia; ^4^ Department of Cardiology, Clinical Sciences, Lund University, Lund, Sweden; ^5^ Arrhythmia Clinic, Skåne University Hospital, Lund, Sweden

**Keywords:** border zone, ECG, epicardial mapping, myocardial ischemia, QT interval and corrected QT interval, ventricular fibrilation

## Abstract

**Background:** Repolarization prolongation can be the earliest electrophysiological change in ischemia, but its role in arrhythmogenesis is unclear. The aim of the present study was to evaluate the early ischemic action potential duration (APD) prolongation concerning its causes, expression in ECG and association with early ischemic ventricular fibrillation (phase 1A VF).

**Methods:** Coronary occlusion was induced in 18 anesthetized pigs, and standard 12 lead ECG along with epicardial electrograms were recorded. Local activation time (AT), end of repolarization time (RT), and activation-repolarization interval (ARIc) were determined as dV/dt minimum during QRS-complex, dV/dt maximum during T-wave, and rate-corrected RT–AT differences, respectively. Patch-clamp studies were done in enzymatically isolated porcine cardiomyocytes. IK(ATP) activation and Ito1 inhibition were tested as possible causes of the APD change.

**Results:** During the initial period of ischemia, a total of 11 pigs demonstrated maximal ARIc prolongation >10 ms at 1 and/or 2.5 min of occlusion (8 and 6 cases at 1 and 2.5 min, respectively) followed by typical ischemic ARIc shortening. The maximal ARIc across all leads was associated with VF development (OR 1.024 95% CI 1.003–1.046, *p* = 0.025) and maximal rate-corrected QT interval (QTc) (B 0.562 95% CI 0.346–0.775, *p* < 0.001) in logistic and linear regression analyses, respectively. Phase 1A VF incidence was associated with maximal QTc at the 2.5 min of occlusion in ROC curve analysis (AUC 0.867, *p* = 0.028) with optimal cut-off 456 ms (sensitivity 1.00, specificity 0.778). The pigs having maximal QTc at 2.5 min more and less than 450 ms significantly differed in phase 1A VF incidence in Kaplan-Meier analysis (log-rank *p* = 0.007). In the patch-clamp experiments, 4-aminopyridine did not produce any effects on the APD; however, pinacidil activated IK(ATP) and caused a biphasic change in the APD with initial prolongation and subsequent shortening.

**Conclusion:** The transiently prolonged repolarization during the initial period of acute ischemia was expressed in the prolongation of the maximal QTc interval in the body surface ECG and was associated with phase 1A VF. IK(ATP) activation in the isolated cardiomyocytes reproduced the biphasic repolarization dynamics observed *in vivo*, which suggests the probable role of IK(ATP) in early ischemic arrhythmogenesis.

## 1 Introduction

Myocardial ischemia leads to a number of proarrhythmic electrophysiological changes in the ventricular myocardium. The typical alteration of action potential duration (APD) in ischemia is its shortening mainly due to the activation of IK(ATP) current (Carmeliet et al., 1999). However, a transient prolongation of APD can be observed as the earliest electrophysiological change in the ischemic conditions, and the cause of this prolongation was established to be the suppression of Ito1 current (Verkerk et al., 1996). This early ischemic APD prolongation is consistent with the findings of the QTc interval lengthening at the first minute of coronary occlusion in pigs (Azarov et al., 2018). However, the porcine ventricular myocardium has been reported to lack the Ito1 current (Li et al., 2003), which warrants further studies of the mechanism of this phenomenon.

During ischemia and reperfusion, the affected myocardium is heterogeneous. A core (or central) ischemic zone and a border ischemic zone can be discerned according to metabolic, electrophysiological, and morphological characteristics of the tissue (Janse et al., 1985; Wilensky et al., 1986; Coronel et al., 1992), though criteria for the discrimination might differ across the experimental settings. Our previous studies have demonstrated that repolarization prolongation can be observed in the border zone of ischemic myocardium, and this prolongation (as well as its ECG correlates) was associated with reperfusion ventricular fibrillation (VF) (Bernikova et al., 2018; Bernikova et al., 2019; Bernikova et al., 2021; Sedova et al., 2022). We hypothesized that similar association can be found at the beginning of an ischemia episode when transient QT interval prolongation is observed.

VF is a potentially lethal complication of myocardial infarction. In experimental settings, VF episodes during acute ischemia are clustered into two subsets, which have been called 1A and 1B phases (Menken et al., 1979). Exploration of the early, phase 1A VF is challenging since relatively short time lapses from ischemia onset to arrhythmia development, and only minor changes in depolarization spread and dispersion of repolarization (DOR) may occur during this period. In humans, where ischemic injury develops severalfold longer than in usual experimental models (Hedström et al., 2009), the early VFs corresponding to the phase 1A, may occur within the first 70 min of ischemia, which is before the first medical contact, and likely represents the major mechanism of sudden cardiac death in the conditions of coronary artery occlusion. Though moderate in magnitude, the transient QT lengthening at the beginning of ischemia might combine with other similarly moderate proarrhythmic changes (e.g., depolarization slowing) and precipitate arrhythmias. However, the role of this early ischemic prolongation of repolarization in arrhythmogenesis remains unclear.

The aim of the present study was to characterize the ischemic APD prolongation at the beginning of acute myocardial ischemia (corresponding to the phase 1A VF) in the porcine model: i) to delineate the probable causes of this phenomenon in the porcine myocardium, specifically to test Ito1 inhibition and IK(ATP) activation as hypothetical causes of the APD changes; ii) to evaluate the duration of repolarization in the core and border ischemic areas and to find out its expression in the parameters of body surface ECG; iii) to test the association of repolarization duration and the appropriate ECG parameters with phase 1A VF incidence.

## 2 Materials and methods

The study conformed to the Guide for the Care and Use of Laboratory Animals, 8th Edition published by the National Academies Press (US) 2011, the guidelines from Directive 2010/63/EU of the European Parliament on the protection of animals used for scientific purposes and was approved by the ethical committee of the Institute of Physiology of the Komi Science Centre, Ural Branch of Russian Academy of Sciences.

### 2.1 *In vivo* animal preparation and experimental protocol

Experiments were performed in 18 pigs (14 males, 4 females, 36 ± 5 kg), anesthetized with zoletil (Virbac S.A., Carros, France, 10–15 mg/kg, i.m.), xylazine (Interchemie, Castenray, Netherlands, 0.5 mg/kg, i.m.) and propofol (Norbrook Laboratories Ltd., UK, 1 mg/kg, i.v.). The animals were intubated and mechanically ventilated. The heart was accessed *via* a midsternal incision, and the temperature in the pericardial cavity was maintained at 37°C–38°C by irrigation with warm saline and heating the room air.

Ischemia was induced by ligating the coronary arteries. In order to avoid an occlusion site-related bias in data interpretation, coronary occlusion was performed at two localizations: left anterior descending artery (LAD), just distal to the origin of the first diagonal branch (*n* = 9) and left circumflex artery (LCX), approximately 2 cm from its origin (*n* = 9). After the ligature placement (not tightened), epicardial leads were positioned (see below), and the thorax was reclosed and left for stabilization for 30 min, after which baseline recordings were done. Then, the ligature was tightened, the thorax was again reclosed, and recordings were repeated during occlusion. The animals were euthanized under deep anesthesia by the intravenous potassium chloride injection either at the end of ischemic episode or immediately after VF development.

### 2.2 Recordings and data processing

Epicardial recordings were done with the aid of a custom-designed system (16 bits; bandwidth 0.05–1,000 Hz; sampling rate 4,000 Hz). Epicardial leads were fixed on a flexible plate, which was placed and sutured to the ventricles to cover both expected ischemic and normal zones. The exact number of epicardial recording leads varied in different individuals (32–64 leads) according to coronary anatomy and surgical accessibility of the regions of interest.

Analysis was performed for the data obtained at baseline (5 min before occlusion) and at the 1st, 2.5, 5th, and 10th min of coronary occlusion in the individual non-averaged beats. Epicardial mapping and ECG data were processed by different investigators (OGB and AST, respectively) in a mutually blinded fashion. In each epicardial lead at each time-point, local activation time (AT) and end of repolarization time (RT) were measured from the QRS onset to the instants of a dV/dt minimum during QRS-complex and dV/dt maximum during T-wave, respectively. Activation-repolarization intervals (ARI) were determined as RT–AT differences. ARIs were rate-corrected with Bazett formula (ARIc = ARI/RR ^1/2^). Dispersion of repolarization was calculated as the difference between maximal and minimal RTs throughout all epicardial leads. Within the mapped area, the zone demonstrating AT delay of more than 5 ms (20%) and the adjacent zone were labeled as core and border ischemic zones, respectively. Maximal and minimal ARIc values, maximal AT value throughout all epicardial leads, and DOR were tested for the association with VF incidence.

In parallel with epicardial mapping, continuous standard 12-lead ECG monitoring (КТ-07-3/12, INCART, St. Petersburg, Russia) was performed (sampling rate 1,028 Hz, dynamic range ±310 mV, 19-bit ADC, amplitude resolution 1.18 μV per bit). Natural and rate-corrected (Bazett formula) QT intervals (QT and QTc, respectively) as well as natural and rate-corrected QTpeak intervals (QTp and QTp_c, respectively) were measured. Minimal QTp and QTp_c (supposedly reflecting the minimal repolarization duration), and maximal QT and QTc (supposedly reflecting the maximal repolarization duration) values throughout all leads at each time-point were taken for analysis. Natural and rate corrected (Bazett formula) Tpeak-Tend intervals (Tpe and Tpe_c) reflecting DOR were calculated as the difference between the instants of the earliest Tpeak and the latest Tend throughout all leads. The intraobserver and interobserver variabilities for repolarization duration measurements were 1.5% (4 ms) and 2.3% (7 ms), respectively.

### 2.3 *In vitro* studies

In the patch-clamp studies we tested inhibition of Ito1 current and activation of IK(ATP) current as the interventions, which might reproduce the changes of APD at the beginning of ischemia.

#### 2.3.1 Cardiomyocyte isolation

Six pig hearts were purchased from the commercial food supplier (Ptitsefabrika Zelenetskaya JSC, Syktyvkar, Komi Republic, Russia). The hearts were quickly excised from the exsanguinated animals and put on ice. Within 5 min, the aorta was cannulated and perfusion with ice-cold calcium-free Tyrode solution was established to stop contractile activity. The composition of the solution (mM) was as follows: (mM): 150 NaCl, 5.4 KCl, 5 MgSO_4_ · 7H_2_O, 10 HEPES; pH 7.2, at 4°C with NaOH. The perfusion lasted for 40 min and took 1.0–1.5 L of the solution. Then, the anterior part of the heart including LAD was dissected (Supplementary Figure S1). The preparation was placed into the bath, the LAD was canulated, and perfusion through the Langendorff system was started with a carbogen-saturated (95% O_2_, 5% CO_2_) modified calcium-free Tyrode solution of the following composition (mM): 120 NaCl, 5.4 KCl, 5 MgSO_4_ · 7H_2_O, 5 sodium pyruvate, 20 glucose, 20 taurine, 1 mg/mL bovine serum albumin; pH 7.2, at 37°C with NaOH. In 15 min, perfusion was switched to the Tyrode solution with the same composition with the addition of enzyme mixture [type II collagenase (0.5 mg/mL, Sigma Aldrich, United States), protease (0.01 mg/mL, Sigma Aldrich, United States) and 20 μM CaCl_2_]. The enzymatic perfusion lasted for 35 min, at the end of which the ventricular tissue was cut with fine scissors into small pieces. The cells were separated from the chunks by filtering through a 200 μm mesh net in Kraft-Brühe solution containing (mM): 30 KCl, 50 glutamic acid, 30 K_2_HPO_4_ · 2H_2_O, 3 MgSO_4_ · 7H_2_O, 0.5 EGTA, 10 glucose, 20 taurine, and 10 HEPES; pH 7.2 with KOH. The cells were stored in Kraft-Brühe solution and used within 6–7 h.

#### 2.3.2 Patch-clamp studies

Whole-cell patch-clamp experiments were performed with a setup based on an Axopatch 200B amplifier (Molecular Devices, San Jose, CA, United States). Ventricular cardiomyocytes were placed into an RC26 experimental chamber (Warner Instruments, Holliston, MA, United States), perfused with a normal Tyrode solution containing (mM): NaCl 138, KCl 5, CaCl_2_ 1.8, MgCl_2_ 5, glucose 10, and HEPES 10, pH = 7.4 at 22°C–24°C with NaOH. Patch pipettes were made of borosilicate glass (Sutter Instrument, Novato, CA, United States) and pulled on a HEKA pipette puller type PIP 6 (HEKA Elektronik GmbH, Reutlingen, Germany). For action potential and IK(ATP) recordings, the pipettes were filled with the internal pipette solution, containing (mM): KCl 140, MgCl_2_ 1, MgATP 4, HEPES 10, Na2GTP 0,03, pH = 7.2 with KOH, and had the tip resistance of 2–3 MΩ. APs were recorded in a current-clamp mode. To study IK(ATP) current we used ramp protocol with 1-s repolarizing voltage ramps from 60 mV to −120 mV every 5 s with and without the application of pinacidil (100 μM). The holding potential was −40 mV. Nifedipine (10 μM) and 4-aminopyridine (2 mM) were added to abolish Ca^2+^currents (I_Ca_) and the transient outward current (I_to_), respectively. Pipette capacitance, whole cell capacitance and access resistance were routinely compensated. The recorded traces were analyzed using Clampfit 9.2 (Molecular Devices, San Jose, CA, United States). IK1 was obtained as Ba-sensitive (leakage corrected) current before the addition of pinacidil. IK(ATP) was obtained by subtracting the IK1 from the total IKir [IK1 + IK(ATP)] current registered in the presence of pinacidil.

#### 2.3.3 Drugs

Unless specified, the chemicals were from Sigma-Aldrich (United States). Type II Collagenase was purchased from Worthington (Lakewood, NJ, United States).

### 2.4 Statistical analysis

Statistical analyses were performed using the SPSS 23 (SPSS, Inc., Chicago, Illinois, United States). Fridman test followed by Wilcoxon test with Bonferroni correction were used for comparison of repeated measurements. The associations between arrhythmic outcomes, ECG and epicardial mapping variables were evaluated in the period 1–10 min of coronary occlusion corresponding to the phase 1A ischemia-related arrhythmias. Linear regression analysis was used for evaluation of the association between myocardial electrophysiological characteristics and ECG parameters. VF incidence in the groups with different coronary occlusion sites was compared with Chi-square test. In order to determine predictors of VF occurrence, logistic regression, Cox regression, and ROC curve analyses were used, followed by the analysis of Kaplan–Meier survival functions. The differences were considered significant at *p* < 0.05.

## 3 Results

### 3.1 *In vivo* studies

**FIGURE 1:**
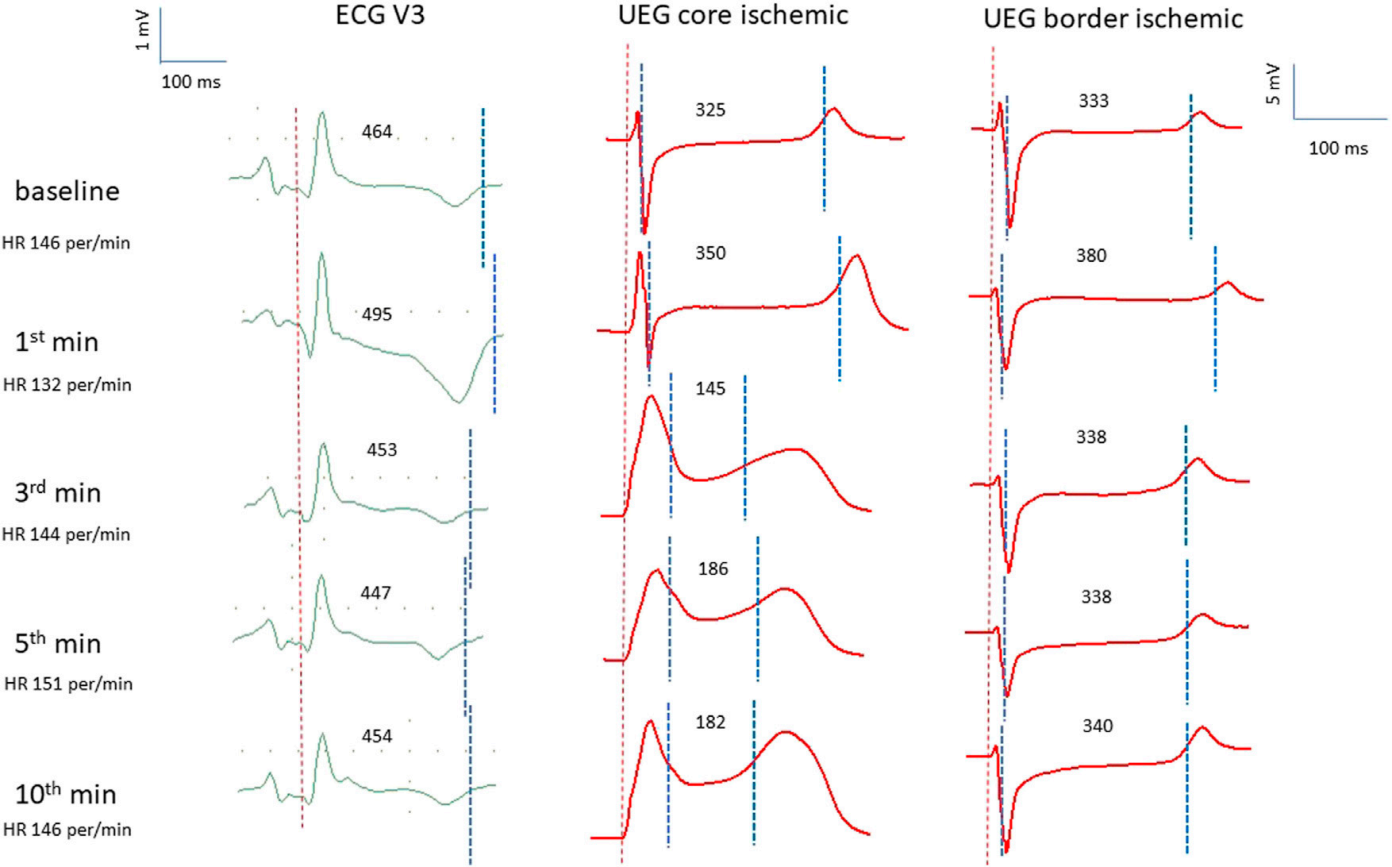
Representative electrocardiograms (ECG lead V3), and epicardial unipolar electrograms (UEG) recorded in the core and border ischemic zones in the same pig at the same time-point. Vertical dotted lines indicate the margins of QT interval in ECGs and the margins of ARI in UEGs. In each UEG, the left and right dotted lines indicate AT and RT, respectively. The individual values of ARI and QT intervals are presented above the tracings. See QT prolongation at the 1^st^ min associated with the prolongation of the ARI in the border zone.

Electrograms recorded in the affected area demonstrated expected ischemic changes including ST-segment elevation and QRS prolongation (Figure 1). In 16 pigs in different epicardial leads during ischemia, a prolongation of ARIc was observed in the core ischemic zone and border ischemic zone [median 72 ms (IQR 44–101 ms), *n* = 7 animals; and median 24 ms (IQR 16–35 ms, *n* = 15 animals, respectively). This ARIc prolongation phase was short-term and lasted until 2.5 min after occlusion onset.

**FIGURE 2:**
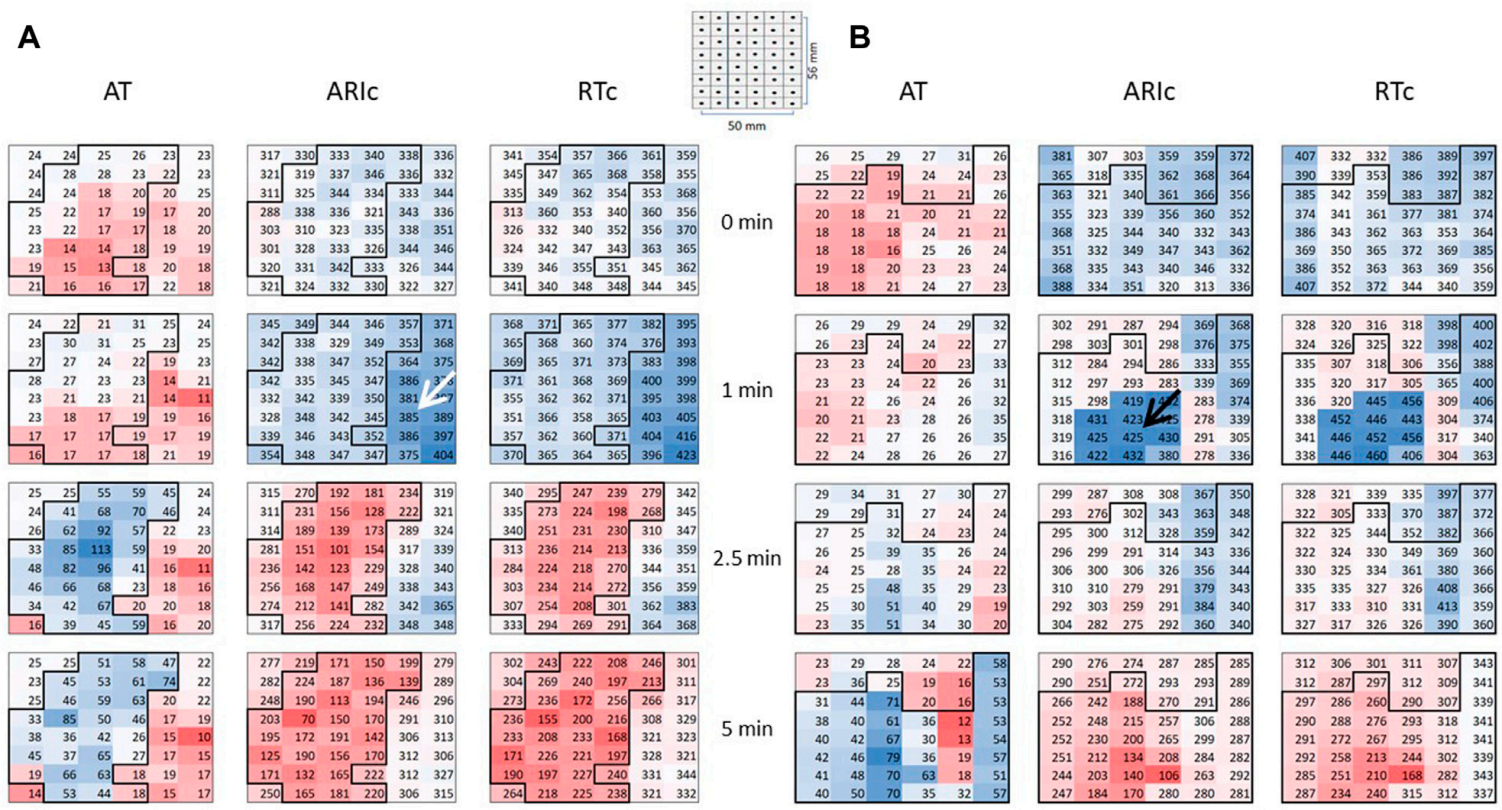
Representative epicardial maps of ATs, ARIc, and RTc in the affected area in two pigs **(A,B)**. Numbers and color labeling (red for short and blue for long) indicate AT, ARIc, and RTc values in the individual leads. RTc values were calculated as RTc = AT + ARIc. See the resemblance of ARIc and RTc maps. Color labeling is the same throughout all time-points in each of six sets of maps (vertical columns). The core ischemic zone was determined on the basis of AT maps (left columns in panels **A** and **B**). To do it, AT values were compared between baseline (0 min) and ischemia (1, 2.5, 5 min). The area with the ischemia-related delays of AT at 1, 2.5, or 5 min is outlined with bold contours and referred to as a core ischemic zones. The surrounding area is identified as a border ischemic zone. Pigs **(A,B)** demonstrated ARIc prolongation in the border (white arrow) and core (black arrows) ischemic zones, respectively. Spatial parameters of the maps are presented in a schematic.

Figure 2 presents two examples of transient and non-uniform ARIc changes within the mapped area. ARIc shortening was consistently observed in the core ischemic zone, though in seven animals a prolongation was observed in a limited number of leads. ARIc in the border zone demonstrated prolongation in 15 animals. The latter effect was short in duration and subsequently transformed into the opposite change (shortening). Different changes in repolarization durations can be also seen in representative electrograms led from the core and border ischemic zones (Figure 1).

**FIGURE 3:**
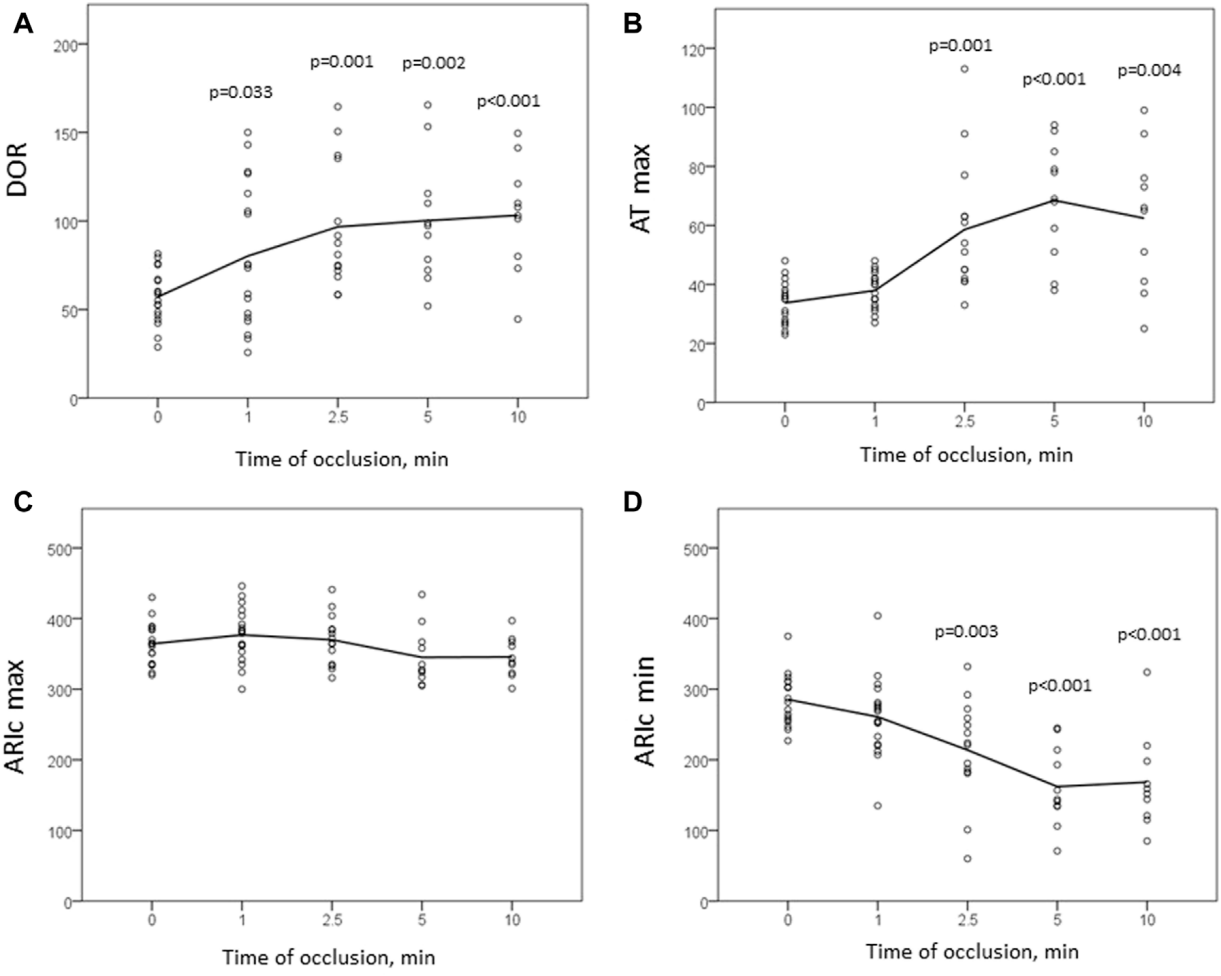
Dynamics of mapping parameters reflecting major prerequisites of reentrant arrhythmias: DOR **(A)**, maximal AT **(B)**, maximal and minimal ARIc (**(C,D)**, respectively). Indicated are *p*-values vs. baseline.

Figure 3 displays time-evolution of major mapping variables during the initial phase of ischemia. The minimal ARIc decreased, whereas the changes of maximal ARIc were statistically insignificant due to different dynamics of maximal ARIc in different animals. A total of 11 pigs demonstrated maximal ARIc prolongation >10 ms at 1 and/or 2.5 min of occlusion (8 and 6 cases at 1 and 2.5 min, respectively). ATs prolonged in the affected area resulting in that the maximal AT increased at 2.5 and 5 min as compared to the baseline. Similar dynamics was observed for DOR.

Nine animals experienced phase 1A VF episode es (1-8 min after occlusion onset). VF incidence did not differ in the animals with LAD (5 episodes) and LCX (4 episodes) occlusion (χ2 0.222 *p* = 0.637) and was not associated with baseline epicardial and body surface ECG parameters. Among all mapping parameters measured during 1–10 min of ischemia (Table 1), only the duration of maximal ARIc throughout all epicardial leads was associated with VF development in univariate logistic regression analysis (Table 2). In linear regression analysis, the maximal ARIc was associated with a maximal QTc interval (B 0.562 95% CI 0.346–0.775, *p* < 0.001, see also similar changes of the border zone ARI and lead V3 QT interval in Figure 1). In the same period (1–10 min), phase 1A VF incidence was associated with maximal QTc interval as a continuous variable in logistic regression analysis (Table 3) and in the ROC curve analysis [AUC 0.736, *p* = 0.027, optimal cut-off 463 ms (sensitivity 0.889, specificity 0.614)]. As a categorical variable, maximal QTc longer than 460 ms predicts VF occurrence in logistic regression analysis with OR 11.556, 95% CI 1.327–100.599 (*p* = 0.027).

**TABLE 1:** Epicardial mapping and ECG characteristics (Mean ± SD) in the studied groups.

Variable	Time, min	Whole group	No phase 1A VF	Phase 1A VF	*p* ^a^
RR, ms	0	534 ± 97	523 ± 97	545 ± 101	0.645
	1	544 ± 141	525 ± 148	562 ± 139	0.730
	2.5	540 ± 111	521 ± 111	575 ± 113	0.397
AT max, ms	0	34 ± 7	33 ± 6	34 ± 8	0.777
	1	38 ± 6	35 ± 5	40 ± 6	0.094
	2.5	59 ± 22	68 ± 22	41 ± 5	0.007
DOR, ms	0	57 ± 15	59 ± 17	56 ± 14	0.656
	1	80 ± 40	67 ± 28	94 ± 47	0.258
	2.5	97 ± 35	99 ± 41	92 ± 25	0.744
Average ARIc, ms	0	330 ± 32	312 ± 23	348 ± 29	0.011
	1	320 ± 39	308 ± 31	332 ± 44	0.297
	2.5	299 ± 39	280 ± 27	333 ± 37	0.009
Minimal ARIc, ms	0	285 ± 36	267 ± 28	304 ± 35	0.031
	1	261 ± 56	264 ± 27	258 ± 77	0.731
	2.5	214 ± 72	184 ± 65	268 ± 51	0.028
Maximal ARIc, ms	0	364 ± 29	352 ± 25	377 ± 29	0.077
	1	377 ± 38	362 ± 41	392 ± 32	0.094
	2.5	370 ± 36	355 ± 32	396 ± 28	0.032
Minimal QTp, ms	0	265 ± 41	246 ± 28	284 ± 44	0.094
	1	261 ± 62	237 ± 54	286 ± 62	0.161
	2.5	242 ± 51	225 ± 46	272 ± 50	0.101
Minimal QTp_c, ms	0	364 ± 42	342 ± 25	386 ± 46	0.024
	1	355 ± 57	327 ± 45	382 ± 57	0.050
	2.5	329 ± 53	312 ± 53	359 ± 44	0.120
Maximal QT, ms	0	331 ± 38	316 ± 31	346 ± 41	0.136
	1	342 ± 53	321 ± 41	362 ± 59	0.258
	2.5	334 ± 46	314 ± 24	370 ± 56	0.023
Maximal QTc, ms	0	455 ± 35	439 ± 23	471 ± 39	0.050
	1	467 ± 39	449 ± 40	485 ± 31	0.050
	2.5	457 ± 39	439 ± 26	488 ± 40	0.018

^a^ Phase 1A VF vs. No Phase 1A VF.

**TABLE 2:** Association between VF incidence and epicardial mapping characteristics in univariate logistic regression analysis (period 1–10 min of coronary occlusion).

Variables	OR	95% CI	*P*
AT max	0.964	0.920–1.011	0.132
DOR	1.009	0.989–1.029	0.391
Average ARIc	1.017	0.999–1.036	0.060
Minimal ARIc	1.004	0.994–1.014	0.401
Maximal ARIc	**1.024**	**1.003–1.046**	**0.025**

Statistically significant associations are in bold.

**TABLE 3:** Association between VF incidence and ECG parameters reflecting longest and shortest durations of repolarization, cardiac cycle length and DOR in univariate logistic regression analysis (period 1–10 min of coronary occlusion).

Variables	OR	95% CI	*P*
RR	1.001	0.995–1.007	0.765
Minimal QTp	1.004	0.990–1.018	0.567
Minimal QTp_c	1.005	0.991–1.019	0.524
Maximal QT	1.013	0.997–1.029	0.113
Maximal QTc	**1.024**	**1.001–1.046**	**0.037**
Tpe	1.006	0.973–1.041	0.719
Tpe_c	1.000	0.975–1.025	0.980

Statistically significant associations are in bold.

**FIGURE 4:**
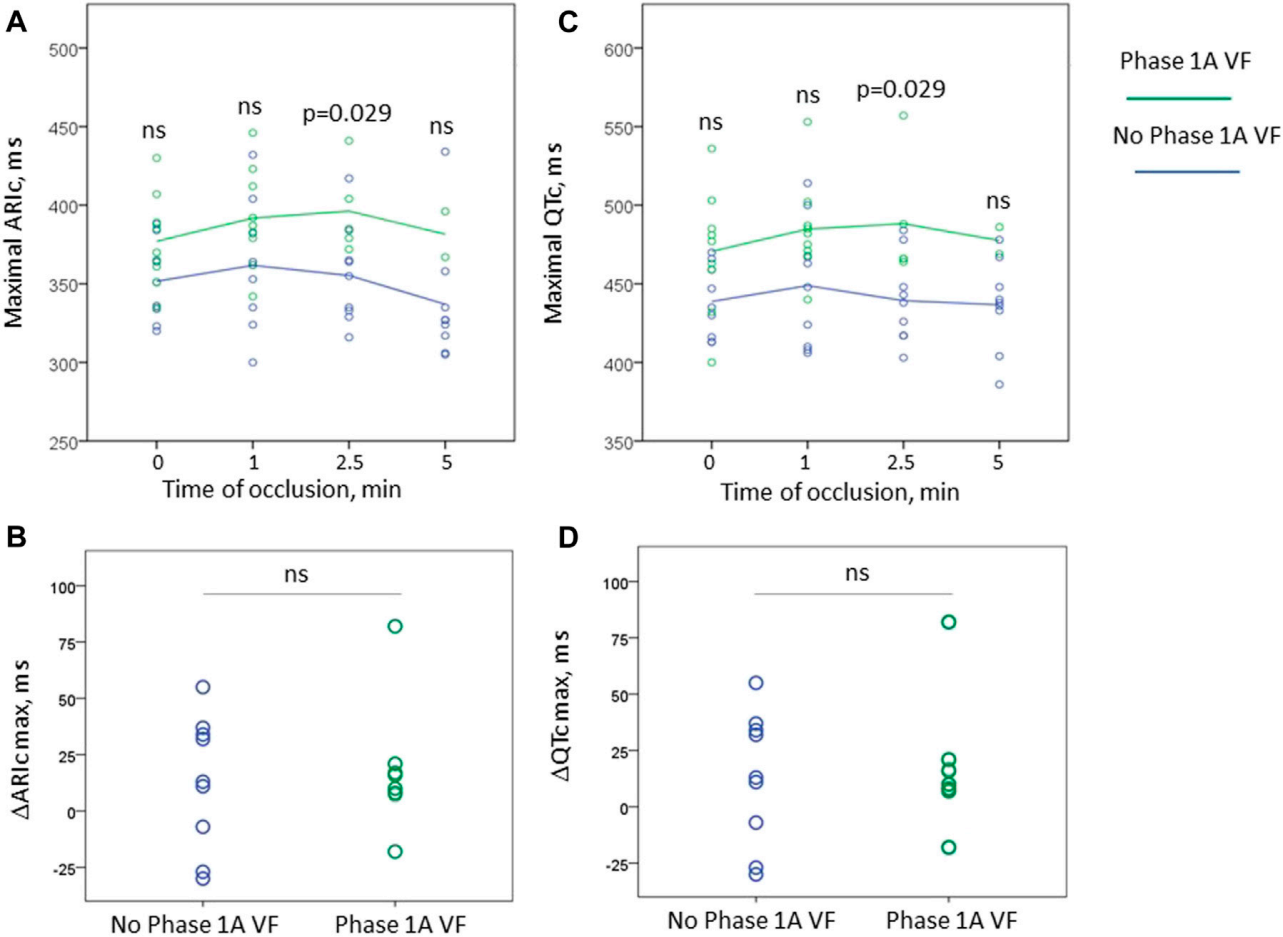
Changes of maximal ARIc (panels **(A,B)**) and maximal QTc intervals (panels **(C,D)**) during first 5 min of occlusion in the animals experiencing phase 1A VF (*n* = 9 at baseline) and no phase 1A VF (*n* = 9). Panels **(A, C)** display absolute values of ARIc and QTc, respectively, plotted vs. time of occlusion. Panels **(B, D)** show the magnitude of maximal change of these variables in respect to baseline. See that only one animal experiencing VF demonstrated shortening of repolarization (negative values in panels **(B, D)**). However, no statistically significant differences between the groups were found in the baseline absolute values and the magnitude of the changes. The only significant difference between VF-resistant and susceptible animals were the absolute values of ARIc and QTc durations at 2.5 min after occlusion onset. ns, non-significant.

Figure 4 displays dynamics of maximal ARIc and maximal QTc intervals separately in the animals who suffered from phase 1A VF and had no phase 1A VF. Neither baseline values nor the magnitude of the increment of both parameters differed between the groups. However, at the end of the period corresponding to the median duration of ARIc prolongation phase (2.5 min) the duration of QTc was significantly longer in the animals suffering from phase 1A VF than in those who experienced no phase 1A VF episodes.

**FIGURE 5:**
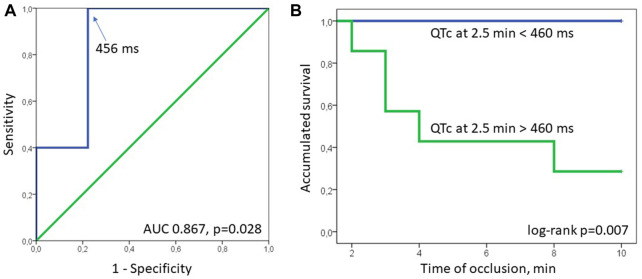
ROC curve (panel **(A)**) and Kaplan-Meier survival (panel **(B)**) analyses. In ROC curve analysis QTc at 2.5 min was associated with phase 1A VF (AUC 0.867, *p* = 0.028) with the optimal cut-off >456 ms (arrow, sensitivity 1.00, specificity 0.778). In Kaplan-Meier survival analysis, phase 1A VF incidence was significantly different (log-rank *p* = 0.007) in pigs having maximal QTc at 2.5 min more and less than 460 ms, respectively.

Neither baseline maximal QTc nor the magnitude of its change by the 2.5th min predict the development of phase 1A VF (OR 1.035 95% CI 0.997–1.075, *p* = 0.075 and 1.005 95% CI 0.971–1.041, *p* = 0.771, respectively). However, the eventual duration of the maximal QTc at 2.5 min, was associated with phase 1A VF in Cox regression analysis (HR 1.024 95% CI 1.002–1.045, *p* = 0.030). In ROC curve analysis, maximal QTc at 2.5 min was associated with phase 1A VF (AUC 0.867, *p* = 0.028) with the optimal cut-off >456 ms predicting VF development with sensitivity 1.00 and specificity 0.778. In Kaplan-Meier survival analysis, phase 1A VF incidence was significantly different (log-rank *p* = 0.007) in pigs having maximal QTc at 2.5 min more and less than 460 ms, respectively (Figure 5).

### 3.2 *In vitro* studies

**FIGURE 6:**
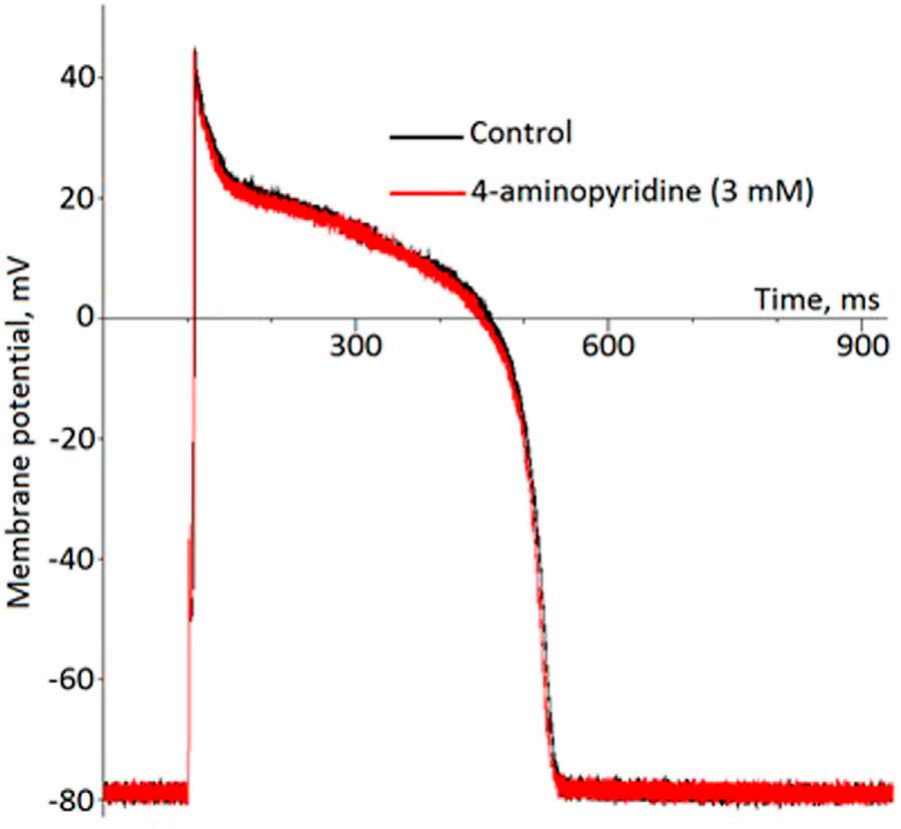
No effect of 4-aminopiridine on the ventricular action potential (representative recordings in the current-clamp whole-cell mode).

**FIGURE 7:**
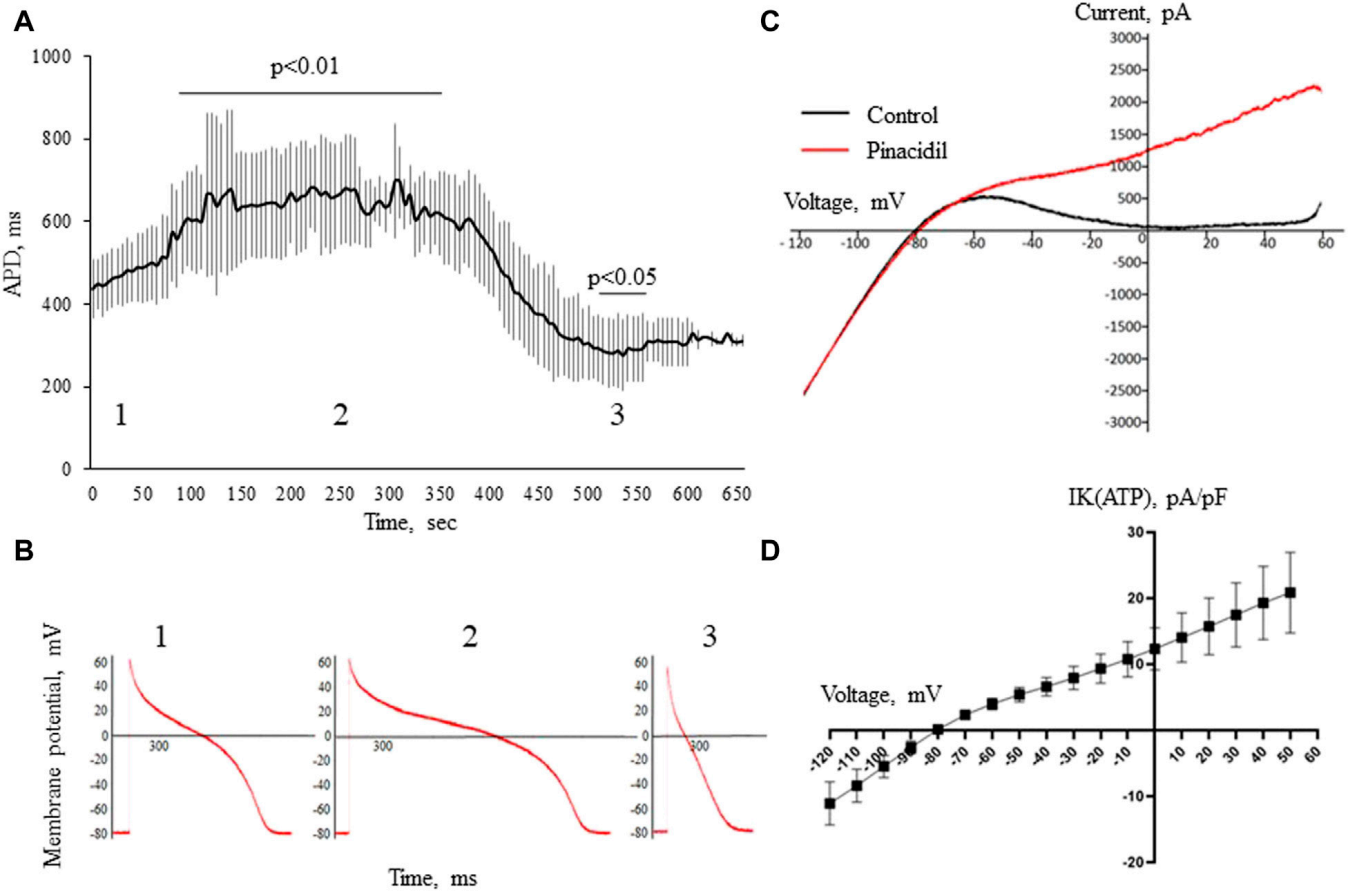
The effects of pinacidil. APD changes during the application of pinacidil in whole-cell configuration current-clamp (panels **(A,B)**). Panel **(A)** shows a biphasic response in APD after the application of pinacidil with the significant prolongation during the early period (55–385 sec, phase 1) and subsequent shortening (475–535 sec, phase 2) as compared to baseline (phase 1). The results are means ± S.E.M. of 6 myocytes from 3 pigs. Panel **(B)** shows the representative AP recordings at baseline (1), 255th (2) and 505th (3) seconds of pinacidil application. Panel **(C)** displays original current recordings in ramp protocol in the control solution and after the application of pinacidil. Panel **(D)** shows a voltage-current curve for IK(ATP) current derived by subtracting the IK1 from the total IKir [IK1 + IK(ATP)] current registered in the presence of pinacidil. The results are means ± S.E.M. of 6 myocytes from 3 pigs.

In the whole-cell current-clamp experiments, 4-aminopyridine, an Ito1 inhibitor did not produce any effects on the AP morphology and duration (Figure 6). On the other hand, pinacidil activated IK(ATP) current and caused a biphasic change in the APD with initial prolongation and subsequent shortening (Figure 7). The prolongation phase developed in 1 min after the pinacidil application and lasted approximately 5 min, after which the steady APD shortening was observed.

## 4 Discussion

### 4.1 Summary of findings

In the present study, we observed transient repolarization prolongation and its subsequent shortening in the border and/or ischemic zone during first minutes of ischemia. This effect was reproduced in isolated cardiomyocytes by the activation of IK(ATP) current. The prolongation of repolarization resulted in the increase in maximal ARIc duration, which in turn was associated with the increase in maximal QTc duration. Prolongation of both maximal ARIc and maximal QTc was associated with the development of the phase 1A VF.

### 4.2 Early ischemic prolongation of repolarization

In our previous study (Azarov et al., 2018), we have observed QTc prolongation at first minutes of the ischemia episode, while the present investigation demonstrated that such a prolongation was associated with the increase in ARIc in the affected area. Interestingly, this ARIc prolongation was observed rather in a border than in a core ischemic area. These data correspond to the previous finding of ARI prolongation in the border area of feline ischemic myocardium (Bernikova et al., 2018). Given the border zone is an at least partly perfused area, the prolongation of repolarization in the border zone might be subject to therapeutic interventions.

Due to the transient nature of this phenomenon, its mechanism is hard to elucidate. Duration of repolarization depends on the balance between repolarizing and depolarizing currents. The most pronounced APD shortening effect in ischemia is due to activation of IK(ATP) (Carmeliet et al., 1999). Verkerk et al. (1996) reported early ischemic APD prolongation in part of cardiomyocytes due to the inhibition of Ito1. On the other hand, the presence and properties of this current in the porcine myocardium is lacking or at least questionable (Lacroix et al., 2002; Li et al., 2003; Schultz et al., 2007). In the present investigation, the application of 4-aminopyridine, which blocks Ito1 produced no effects on APD, that supports the cited above findings. On the other hand, activation of IK(ATP) by pinacidil induced the transient APD prolongation followed by the steady APD shortening with temporal dynamics quite similar to that observed *in vivo*. It suggests that IK(ATP) could play its role not only in the APD shortening but also in the APD prolongation. This observation needs further exploration since it is obvious that the repolarizing current cannot directly increase APD, but indirect effects are probable especially when IK(ATP) activation did not still reach its maximal extent. Such indirect effects might include metabolic changes, the increase of the electromotive force for calcium current etc.

Whatever the exact ionic mechanism is involved in APD prolongation response to ischemia, it could be noted that absolutely or relatively prolonged repolarization is usually associated with relatively mild ischemic conditions (Smith et al., 2012), including non-ST-segment elevation myocardial infarction (vs. ST-segment elevation myocardial infarction) (Henrie et al., 2017) and chronic intermittent hypoxia (Morand et al., 2018). Local QTc prolongation indicates the presence of viable myocardium in the affected areas (Ieva et al., 2016). Furthermore, QTc prolongation has been shown to be an *early* marker of myocardial ischemia at admission (Kenigsberg et al., 2007; Jiménez-Candil et al., 2008) or in stress-tests (Guideri et al., 1995; Arab et al., 2000; Vora et al., 2003).

Our data presented in the Figure 2 demonstrate that ARIc prolongation in either core ischemic or border ischemic zones was followed by the typical ischemic shortening of repolarization, which evidences that this region was indeed affected. On the other hand, most frequently ARIc (or APD) prolonged in the regions beyond but close to the area of the typical ischemic AT delay and only at the very beginning of ischemia [or at the beginning of *in vitro* IK(ATP) activation]. Collectively these findings suggest the observed repolarization prolongation was a signature of relatively mild ischemic conditions.

### 4.3 Arrhythmogenesis at early ischemia

The present study demonstrated that prolongation of repolarization at first minutes of ischemia were associated with phase 1A VF. Such an association is usually interpreted as a consequence of the increased DOR resulting from a combination of the prolonged and shortened repolarization durations outside and inside the affected area, respectively (Kuo et al., 1983). However, there are several reasons to exclude DOR as the link between VF development and ARIc prolongation in this setting. i) DOR was tested as a VF predictor in logistic regression analysis and no significant associations were found (though low statistical power should be taken into account). ii) The previous studies (Tsvetkova et al., 2020; Tsvetkova et al., 2021) showed that DOR increases much later during the ischemic episode and contributes to the development of phase 1B VF. iii) On the other hand, APD prolongation can have an independent effect on arrhythmogenesis that can be even superior to that of DOR (Bernikova et al., 2019; Bernikova et al., 2021). Probably, it is related to the fact that the prolonged APD facilitates early afterdepolarizations (Dutta et al., 2016) or phase 2 reentry (Yan et al., 2004) that can trigger reentrant arrhythmias. However, these explanations warrant direct testing.

The observed proarrhythmic ARIc prolongation was expressed in the corresponding change of QTc interval, and the prolonged QTc interval was associated with phase 1A VF development in a manner similar to ARIc. It is obvious, that the duration of the ischemic QTc interval can be presented as a sum of its baseline value and the magnitude of change caused by ischemia. We tried to evaluate both contributors, but neither baseline QTc duration, nor the magnitude of its change discriminated between phase 1A VF-susceptible and phase 1A VF-resistant animals. Only the resulting value of QTc at the beginning of the ischemic episode (2.5 min) was associated with the arrhythmic outcomes.

Prediction of the earliest VF episodes (1–2.5 min) is challenging due to the fact that different individuals demonstrated different time-courses of electrophysiological changes during this short period. That is why we used the time-point of 2.5 min (the end of repolarization prolongation phase) for the survival analysis. However, we believe that prolonged repolarization was also related to the arrhythmogenesis in the period of 1–2.5 min. In order to take these earliest events into account, we tested VF incidence in the period 1–10 min for the association with QTc measured at a time-point preceding the outcome. In support of our idea, we found the significant association between the VF development and the maximal QTc (both continuous and categorical variables) in the logistic regression analysis throughout the period (1–10 min).

### 4.4 Limitations

Recording of the cardiac electrograms was done only by epicardial contact mapping, while significant electrophysiological changes can occur in the midmyocardial layers. However, these intramural changes largely concern depolarization, not repolarization (Azarov et al., 2019; Tsvetkova et al., 2020), and this limitation would hardly modify the major findings of the present study. Snapshot recordings were done at certain time-points, which limited determination of temporal characteristics including the duration of the ARIc prolongation phase. The short snapshot duration did not permit processing the long time windows, and the measurements were done in the individual complexes. A direct determination of the area at risk localization in respect to epicardial recording leads was not possible for technical reasons, and the ischemic and border zones were discriminated on the basis of the presence or absence of the ischemia-induced activation time delay. Due to this limitation, the data on spatial differentiation between the two zones should be interpreted cautiously. Anesthesia *via* the influence on autonomic tone might modify the repolarization durations in the ventricles, which should be taken into account when awake subjects are considered. Though the statistically significant differences and associations were revealed, it is of note that the statistical power was relatively low.

## 5 Conclusion

In the experimental setting, the transiently prolonged repolarization during the initial period of acute ischemia was expressed in the prolongation of the maximal QTc interval in the body surface ECG and appeared to be a proarrhythmic factor independent of DOR and AT delay. Most often, though not exclusively, this prolongation of repolarization was found in the border ischemic zone beyond the area with most pronounced electrophysiological alterations. The fact that these *in vivo* changes were reproduced *in vitro* by IK(ATP) current activation implies a novel mechanism for the early arrhythmogenic alteration in the ischemic myocardium.

## Data Availability

The raw data supporting the conclusion of this article will be made available by the authors, without undue reservation.
