# Supplementary material for: Prolonged repolarization in the early phase of ischemia is associated with ventricular fibrillation development in a porcine model
Source: Front Physiol. 2023 Jan 23;14:1035032. doi: 10.3389/fphys.2023.1035032 (PMC9899978; doi:10.3389/fphys.2023.1035032)
Supplement: Supplementary file 1 [file Image1.pdf]

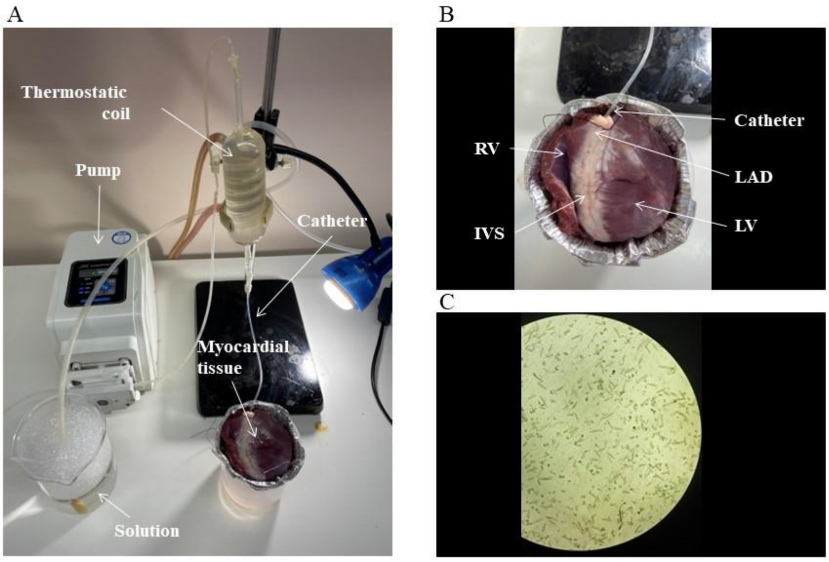

Supplementary Figure 1. Enzymatic isolation of porcine cardiomyocytes. Panel A: Setup for Langendorff tissue perfusion. Panel B shows the cannulated and perfused anterior part of right (RV) and left (LV) ventricles and interventricular septum (IVS) via the left descending coronary artery (LAD). Panel C shows enzymatically isolated porcine cardiomyocytes.
